# Adenoids and Hypertrophied Faucial Tonsils, as Portals of Infection—a Plea to the General Practitioner for Their Early Recognition and Removal

**Published:** 1902-11

**Authors:** P. M. Payne

**Affiliations:** Brownwood, Texas, Former Resident Surgeon of the Eye, Ear, Nose and Throat Hospital, New Orleans; late Oculist and Aurist of American Hospital and Mexican National Railroad, City of Mexico


For Texas Medical Journal.

### Adenoids and Hypertrophied Faucial Tonsils, as Portals of Infection—A Plea to the General Practitioner for Their Early Recognition and Removal.

BY P. M. PAYNE, M. D., BROWNWOOD, TEXAS,
Former Resident Surgeon of the Eye, Ear, Nose and Throat Hospital, New Orleans; late Oculist and Aurist of American Hospital and Mexican National Railroad, City of Mexico.

It has been said that if a man teaches one truth or helps to implant in the minds of his fellow beings one absolutely worthy thought, his life has been worth while.

In writing this short article I shall attempt to teach nothing new, though I may impress a truth on the readers of the "Red-Back."

The ear, nose and throat literature of today abounds with articles from men of all nationalities, on the supreme importance of the removal of the hypertrophied tonsils (faucial and pharyngeal) as early as they are observed. Being essentially a disease of childhood, they are often passed over by the general practitioner, who is not always prepared to operate on them, though he recognizes the importance of doing so. Especially is this the case in hypertrophy of the pharyngeal tonsil (adenoids—Luschka's gland) the operation for which, while simple, requires that the operator shall be thoroughly acquainted with it and have the proper assistance.

But, as to the tonsils being portals of infection, no writer or clinician of today disputes; but, on the contrary, all concur in the belief. The normal tonsil possibly plays some protective part in the economy of the system, but it is very often diseased and becomes a point of distribution for toxic poisons, which are taken up into the system. This can be said of the exanthemata, especially if we concede them to be diseases produced by germ life, the germ resting for a time in the tonsillar crypts and passing into the system, or possibly being the direct cause of the infection, rather than by their toxins. So might we have a typhoid infection, as we most surely do have a rheumatic affection of the tonsils, which will yield only to an anti-rheumatic treatment.

I wish to speak principally of the pharyngeal tonsil and the still greater importance of watching for the symptoms indicative of it. As before stated, it is a condition of childhood and will come first under the notice of the general practitioner; for often the child will be only a year old, possibly younger. However, the majority of the cases will be older, and as the child grows up to four or five years of age, it will be seen to be an habitual "mouth breather" and have repeated "colds," for which it has been given everything in the materia medica, until the parents take it to some "catarrh specialist" who writes a prescription for some nose wash and gives a powder to be sniffed up the nose. The child has had its faucial tonsils removed and the doctor feels like he has done all he can do, for a successful adenotomy requires a general anesthetic (the family physician cannot do it alone). But let me say here that every man who has a license to practice medicine in the United States ought to know how to diagnosticate adenoids, and if he is not prepared to operate for their removal, should insist, and insist again, on having the child carried to some one who is prepared. for I sincerely believe that many cases of catarrhal pneumonia, croup, diphtheria and scarlatina, not to mention thousands of cases of suppurative middle-ear trouble, and consequent deafness, could be obviated by the timely removal of the adenoids and faucial tonsils. Nor is this all. How many school children do we see when they grow up to be ten or twelve year old who, having been heretofore bright and studious, lose interest in their books, go to the foot of their classes and stand around listless and gaping? Examine one of these children and we find an hypertrophied bunch of glands entirely plugging up the posterior nares, impinging on the eustachian tubes, making it impossible for him to breathe, save through his mouth, and when he catches a cold, unable to hear a watch tick ten inches away. About this stage of the disease the upper teeth stick out like squirrel teeth, and he "talks through his nose" (just what he doesn't do, for his nose is entirely stopped up, only on rare occasions). He is now taken to the dentist, who should always advise the removal of the adenoids, as well as treat the reduction of the arched palate, by mechanical means.

In one year's service as resident surgeon of the Eye, Ear, Nose and Throat Hospital, of New Orleans, and two years' special work in the City of Mexico, the writer has personally operated and assisted in these operations over two hundred times. The anesthetic always being, in cases under twelve or fourteen years of age, bromide of ethyl (Merck's), and for older persons, cocaine. Some operators recommend operations by force on young children, but I denounce this as a brutal and heartless procedure, not to mention the fact that it is nearly impossible to clear the naso-pharynx, especially the side walls, without the aid of an anesthetic.

Under bromide of ethyl, the little patient sits upright, the head being supported by an assistant, who may give the anesthetic, though I have given it myself (in private practice) and then removed the adenoids before consciousness was regained. (This last was where I had only an untrained assistant.)

The operation need not take over five to ten minutes, and in Dr.

DeRoaldes' clinic we have performed as high as ten in a little less than one hour.

Just one word further about bromide of ethyl. For years Dr. DeRoaldes fought the fight almost alone in the United States for bromide of ethyl, but current literature shows a decided increase in the advocates of this drug as an anesthetic. The writer claims the honor of being the first to have used it in the City of Mexico, in the operation of adenotomy (September, 1898), and in all the cases that have come under my observation, both in hospitals and private practice, I have never yet seen any untoward effects from its use.

In conclusion, I wish to say, that this little article is written expressly for the busy general practitioners and is intended as a plea to them to look more closely into the cases of "croup and colds" in their practice among children. If I can impress this one point on their minds I shall have done a service to the poor little fellows whose lives, at the very best, are a struggle for existence.

